# WY14643 Increases Herpesvirus Replication and Inhibits IFNβ Production Independently of PPARα Expression

**DOI:** 10.1128/spectrum.02337-22

**Published:** 2023-01-30

**Authors:** Lili Tao, Phillip Dryden, Alexandria Lowe, Guoxun Wang, Amritha Achuthkumar, Tyron Chang, Tiffany A. Reese

**Affiliations:** a Department of Immunology, University of Texas Southwestern Medical Center, Dallas, Texas, USA; b Department of Microbiology, University of Texas Southwestern Medical Center, Dallas, Texas, USA; Oklahoma State University

**Keywords:** MHV68, PPAR, PPAR agonist, herpesvirus, type I interferon

## Abstract

Peroxisome proliferator activated receptor (PPAR) agonists are commonly used to treat metabolic disorders in humans because they regulate fatty acid oxidation and cholesterol metabolism. In addition to their roles in controlling metabolism, PPAR agonists also regulate inflammation and are immunosuppressive in models of autoimmunity. We aimed to test whether activation of PPARα with clinically relevant ligands could impact gammaherpesvirus infection using murine gammaherpesvirus-68 (MHV68, MuHV-4). We found that PPAR agonists WY14643 and fenofibrate increased herpesvirus replication *in vitro*. *In vivo*, WY14643 increased viral replication and caused lethality in mice. Unexpectedly, these effects proved independent of PPARα. We found that WY14643 suppressed production of type I interferon after MHV68 infection *in vitro* and *in vivo*. Taken together, our data indicate that caution should be employed when using PPARα agonists in immuno-metabolic studies, as they can have off-target effects on viral replication through the inhibition of type I interferon production.

**IMPORTANCE** PPAR agonists are used clinically to treat both metabolic and inflammatory disorders. Because viruses are known to rewire host metabolism to their own benefit, the intersection of immunity, metabolism, and virology is an important research area. Our article is an important contribution to this field for two reasons. First, it shows a role for PPARα agonists in altering virus replication. Second, it shows that PPARα agonists can affect virus replication in a manner independent of their predicted target. This knowledge is valuable for anyone seeking to use PPARα agonists as a research tool.

## INTRODUCTION

Viruses manipulate host cellular machinery, including metabolic pathways, to aid their own replication or to suppress host immune defenses. For instance, many human herpesviruses rewire glycolysis, glutaminolysis, and fatty acid synthesis to promote nucleotide synthesis and lipogenesis, allowing the host cell to sustain the high energy demands during viral infection and replication (1–5). Additionally, recent studies show that metabolic products inhibit type I interferon production downstream of both RNA and DNA sensing pathways (6, 7), suggesting the possibility that metabolically active pharmaceuticals might affect virus infection.

To determine whether and how such compounds might impact virus infection and the immune system, we initially focused on the possible interactions of herpesvirus infection with compounds that target peroxisome proliferator activated receptors (PPARs). We chose to study herpesvirus/PPAR interactions because activation of all three PPAR isoforms—PPARα, PPARβ/δ, and PPARγ—profoundly impacts cellular metabolism and inflammatory signaling cascades (8). PPARα activation induces fatty acid oxidation and lowers intracellular lipid levels. PPARγ receptors are primarily expressed in adipose tissue and control lipogenesis and insulin sensitivity. PPARβ/δ receptors also orchestrate fatty acid oxidation, particularly in muscle (8). Consistent with their roles in cellular fatty acid metabolism, activation of PPARs by agonists such as fibrates (PPARα) and thiazolidinediones (PPARγ) has demonstrated clinical efficacy in treating metabolic disorders in humans (9–11). Importantly, fatty acid metabolism has been shown to regulate host-virus interaction and contribute to determining the outcome of infection (12, 13).

Moreover, PPARs regulate inflammation, and PPAR agonists are used clinically to reduce inflammation in atherosclerosis, diabetes, neurodegenerative diseases, and autoimmune diseases (10). Working through diverse pathways, PPAR agonists repress NF-κB and AP-1 DNA binding, regulate nitric oxide production, inhibit dendritic cell maturation, reduce cytokine expression by effector T cells, and inhibit leukocyte recruitment to sites of inflammation, all of which are known key regulators of cellular antiviral response (14, 15). Collectively, this evidence highlights the potential roles of PPARs in shaping immune responses against DNA viruses.

Conversely, it is known that herpesviruses manipulate host cell metabolism during infection to promote viral replication and chronic infection (16, 17), including through the induction of peroxisomes (18, 19). A recent report found that HCMV induces peroxisome biogenesis to enhance plasmalogen synthesis, which is required for efficient HCMV envelopment (19). Herpesviruses also encode viral proteins that target peroxisomes, suggesting that modulation of peroxisomal function is important for these viruses (20–22).

Despite their immunoregulatory functions, our understanding of the effects of PPAR agonists on infectious disease outcomes remains incomplete. There are contradictory reports suggesting that synthetic agonists or dietary lipids improve or impair resistance to pathogen challenge, and the molecular mechanisms of PPAR-mediated immunoregulation during infection remain elusive (23–27).

Thus, we sought to test the hypothesis that PPAR agonists could alter host-herpesvirus interactions. We evaluated agonists of PPARα, PPARβ/δ, and PPARγ and found that the compounds WY14643 and fenofibrate (agonists of PPARα) produce strong proviral effects. Treatment with these compounds increased the replication of herpesviruses in two different taxa. Consistent with our hypothesis, this effect initially seemed dependent on PPARα expression. However, anomalous data led us to carefully control for mouse genetic background and microbiome and revealed that these proviral effects occur independently of PPARα. We further demonstrate that WY14643 treatment in macrophages reduced interferon (IFN)-β and interferon stimulated gene (ISG) expression after virus infection. WY14643’s proviral and interferon-suppressing effects were seen *in vivo* as well, significantly increasing MHV68 replication and animal mortality and decreasing ISG transcription.

## RESULTS

### PPARα agonists promote herpesvirus replication *in vitro*.

To examine the effects of PPAR activation on DNA virus infection, we used murine gammaherpesvirus-68 (MHV68) as our model. MHV68 readily infects mice and undergoes phases of infection similar to human gamma-herpesviruses such as Kaposi’s sarcoma-associated herpesvirus (KSHV) and Epstein Barr virus (EBV) (28, 29). The replication of MHV68 was measured in bone marrow-derived macrophages (BMDMs), as this virus infects and replicates in macrophages (as well as B cells and dendritic cells) *in vivo* (28, 30).

**FIG 1:**
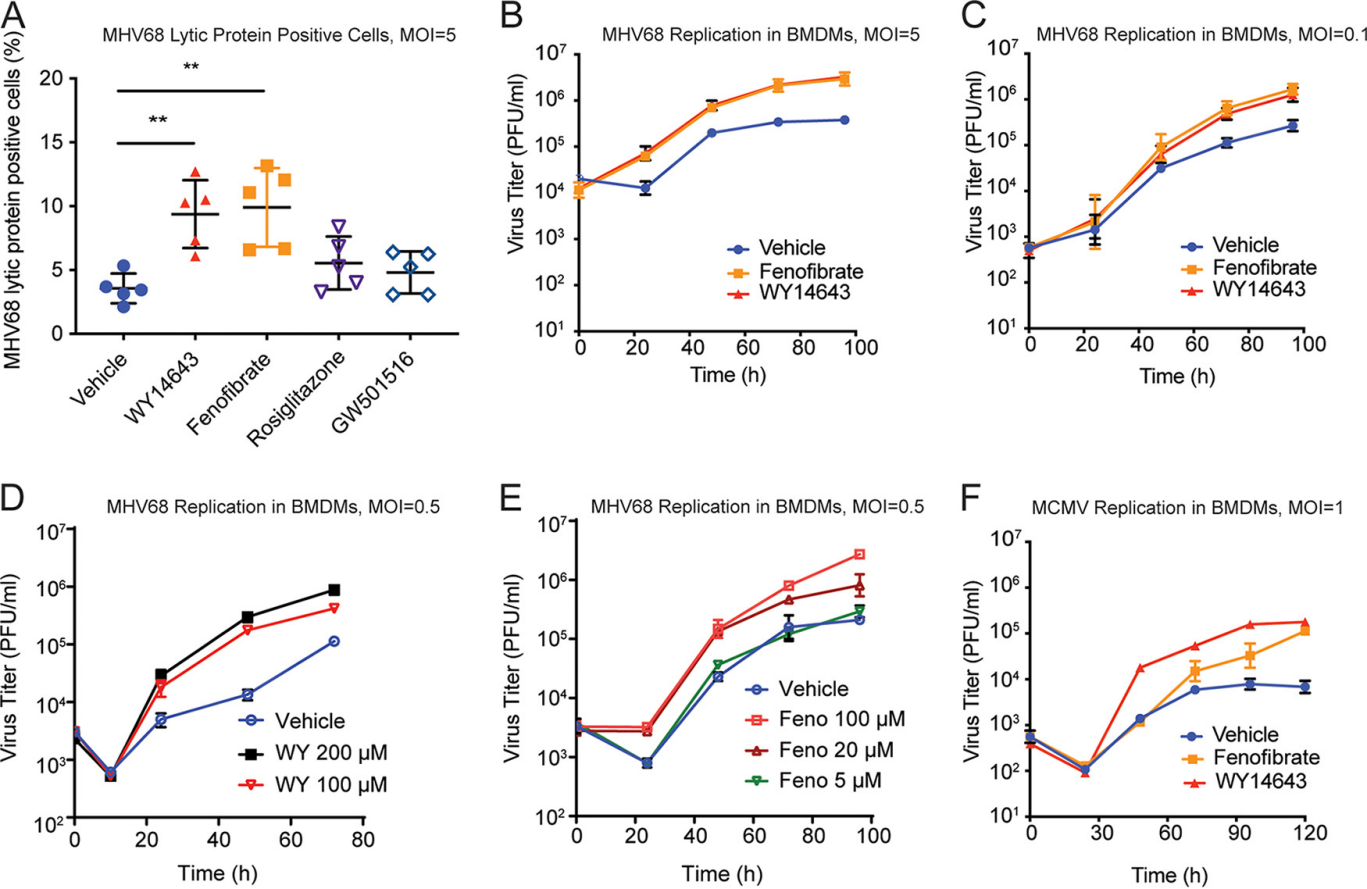
PPARα agonist treatment increases herpesvirus replication *in vitro*. (A) Percentage of BMDMs expressing MHV68 lytic proteins after 24 h of infection and treatment with PPAR agonists. BMDMs from C57BL/6J mice were pretreated for 16 h with WY14643 (200 μM), fenofibrate (50 μM), rosiglitazone (1 μM), or GW501516 (100 nM). Cells were infected with MHV68 at MOI = 5. The percentage of cells expressing MHV68 lytic proteins was measured with flow cytometry using a polyclonal antibody 24 h after infection. The dots represent individual experiments. Data represent the mean ± SD from 5 independent experiments. (B–C). Growth curves of MHV68 in macrophages isolated from C57BL/6J mice after pretreatment with vehicle control (DMSO), WY14643 or fenofibrate. Cells were infected with MHV68 at MOI = 5 (B) or MOI = 0.1 (C). Virus was quantitated by plaque assay on 3T12 cells. The data represent the mean ± SD from 5 independent experiments. D–E. Growth curves of MHV68 in macrophages isolated from C57BL/6J mice with different doses of WY14643 (D) or fenofibrate (E). Virus was quantitated by plaque assay on 3T12 cells. The data represent the mean ± SD from 3 independent experiments. (F) Growth curves of MCMV in BMDMs isolated from C57BL/6J mice. After pretreatment with vehicle, WY14643, or fenofibrate, cells were infected with MCMV at MOI = 1. The data represent the mean ± SD from 2 independent experiments. FACS data were found to be normal and analyzed with one-way ANOVA and Tukey’s multiple-comparison tests. Data are all shown as mean ± SD; *, *P* < 0.05; **, *P* < 0.01; ***, *P* < 0.001; ****, *P* < 0.0001.

The effects of PPAR activation on MHV68 replication were examined by treating BMDMs with agonists for PPARα (fenofibrate or WY14643), PPARβ/δ (GW501516), or PPARγ (rosiglitazone) prior to infection. After infection, we quantified viral replication with flow cytometry, staining for the expression of lytic viral proteins on the surfaces of infected cells (31). We found that PPARα agonists fenofibrate and WY14643 both increased expression of lytic viral proteins on infected macrophages (Fig. 1A). However, GW501516 and rosiglitazone had no effect on MHV68 replication, indicating that PPARβ/δ and PPARγ agonists do not regulate MHV68 replication in BMDMs at the doses commonly used in the literature to stimulate PPARβ/δ and PPARγ (Fig. 1A). We confirmed these effects of WY14643 and fenofibrate with viral growth curves at high and low high multiplicity of infection (MOI), both of which showed increased MHV68 replication in macrophages treated with fenofibrate or WY14643 compared to untreated cells (Fig. 1B, C). Moreover, effects of PPARα agonists fenofibrate and WY14643 increased with increasing dose (Fig. 1D, E) Thus, treatment with PPARα agonists increases MHV68 replication.

To determine if PPARα agonists affect replication of other herpesviruses that infect BMDMs, we tested whether WY14643 or fenofibrate would increase replication of murine cytomegalovirus (MCMV), a betaherpesvirus that also readily infects BMDMs. We found that replication of MCMV was also increased by these treatments (Fig. 1F), indicating that these effects of PPARα agonists apply to at least one other subfamily of herpesvirus.

**FIG 2:**
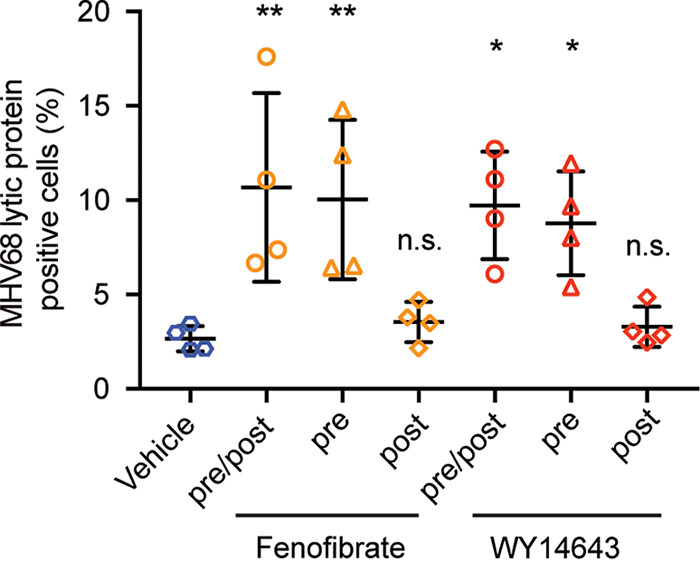
Increased MHV68 replication by WY14643 requires pretreatment. Percentage of BMDMs expressing MHV68 lytic proteins after 24 h of infection with different programs of agonist treatment. BMDMs from C57BL/6J mice were infected with MHV68 at MOI = 5. The cells treated with agonists for 16 h before infection, treated after infection, or both. The percentage of cells expressing MHV68 lytic protein was measured as previously described by flow cytometry. Dots are individual experiments. The data represent the mean ± SD from 4 independent experiments. Data are all shown as mean ± SD; *, *P* < 0.05; **, *P* < 0.01; ***, *P* < 0.001; ****, *P* < 0.0001.

We wondered if PPARα agonist effects could promote virus replication even if cells were treated with agonist after infection, or if the effects of agonist required pretreatment. To test this, we compared three different treatment protocols. We pretreated, as before, with PPARα agonists overnight and replaced agonist in the media following infection with MHV68 (pre/post). We compared this with pretreatment only (pre) or post treatment only (post). We found that pretreatment with PPARα agonists, fenofibrate, and WY14643 was required to increase MHV68 replication (Fig. 2). We found no increase in virus replication when cells were treated postinfection with agonist. We also found that pretreatment alone was sufficient to increase virus replication. These data suggest that PPARα agonists alter the cellular environment prior to infection in such a way that enhances virus replication.

### WY14643 increases virus replication independently of expression of PPARα.

**FIG 3:**
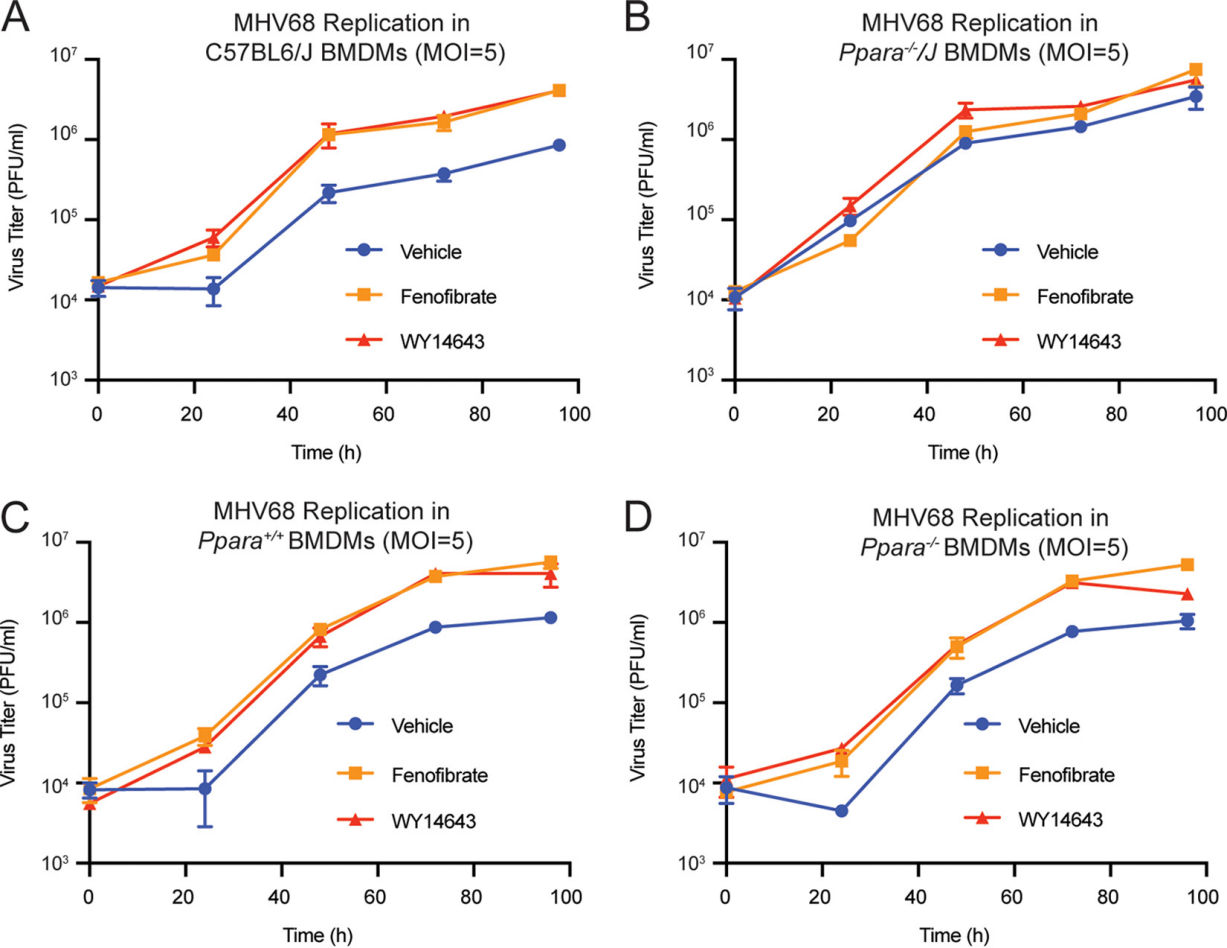
Littermate controls reveal that WY14643 and fenofibrate increase viral replication independently of PPARα *in vitro.* (A–B) Growth curves of MHV68 in BMDMs isolated from either C57BL/6J (A) or *Ppara^−/−^/J* (B) mice. Cells were pretreated with vehicle control, WY14643, or fenofibrate and then infected (MOI = 5). Viral growth was quantitated by plaque assay on 3T12 cells. Data represent the mean ± SD of 5 independent experiments. (C–D) Growth curves of MHV68 in BMDMs isolated from littermate-controlled *Ppara^+/+^* (C) or *Ppara^−/−^* (D) mice. Cells were pretreated with vehicle control, WY14643, or fenofibrate and then infected (MOI = 5). Viral growth was quantitated by plaque assay on 3T12 cells. Data represent the mean ± SD of 5 independent experiments.

We next questioned whether the effects of PPARα agonists WY14643 and fenofibrate depend on expression of PPARα. To answer this question, we initially performed viral growth curve experiments in BMDMs isolated from C57BL/6J or *Ppara^−/−^* mice obtained from Jackson Laboratories (here denoted “*Ppara^−/−^/J*”). In these experiments, the fenofibrate and WY14643 increased viral replication (Fig. 3A). These effects were abolished in the *Ppara^−/−^/J* BMDMs (Fig. 3B). This suggested that their proviral activity involves their canonical role as PPARα agonists. However, we noted an increase in virus replication in vehicle-treated *Ppara^−/−^/J* macrophages relative to control macrophages from C57BL/6J mice (Fig. 3B), which was unexpected.

Questioning whether this was due to differences in mouse genetic background or microbiome differences between knockout and C57BL/6J mice, we generated littermate-controlled mice by crossing C57BL/6J mice with *Ppara^−/−^/J* mice to obtain heterozygous offspring. These heterozygous mice were interbred to yield both knockout (“*Ppara^−/−^*”) and wild-type (“*Ppara^+/+^*”) animals that were used for BMDM generation. When we compared viral replication in these littermate-controlled macrophages, we observed no baseline difference in virus replication between the genotypes in the vehicle group (Fig. 3C, D). However, the effects of fenofibrate and WY14643 remained intact in the littermate-controlled knockout BMDMs; agonist treatment was associated with increased virus replication in PPARα-deficient cells just as in wild-type cells (Fig. 3D). This suggests that fenofibrate and WY14643 increase virus replication independently of PPARα expression.

At first, our *in vitro* data suggested that the proviral effects of fenofibrate and WY14643 depend on PPARα. However, this seems to be an artifact of different mouse genetic backgrounds or microbiome differences between the C57BL/6J and knockout colonies; when we used littermate controls, this dependency vanished. This indicates that WY14643 increases MHV68 replication independently of its canonical role as agonists of PPARα.

### WY14643 suppresses the interferon response by reducing type I IFN production.

**FIG 4:**
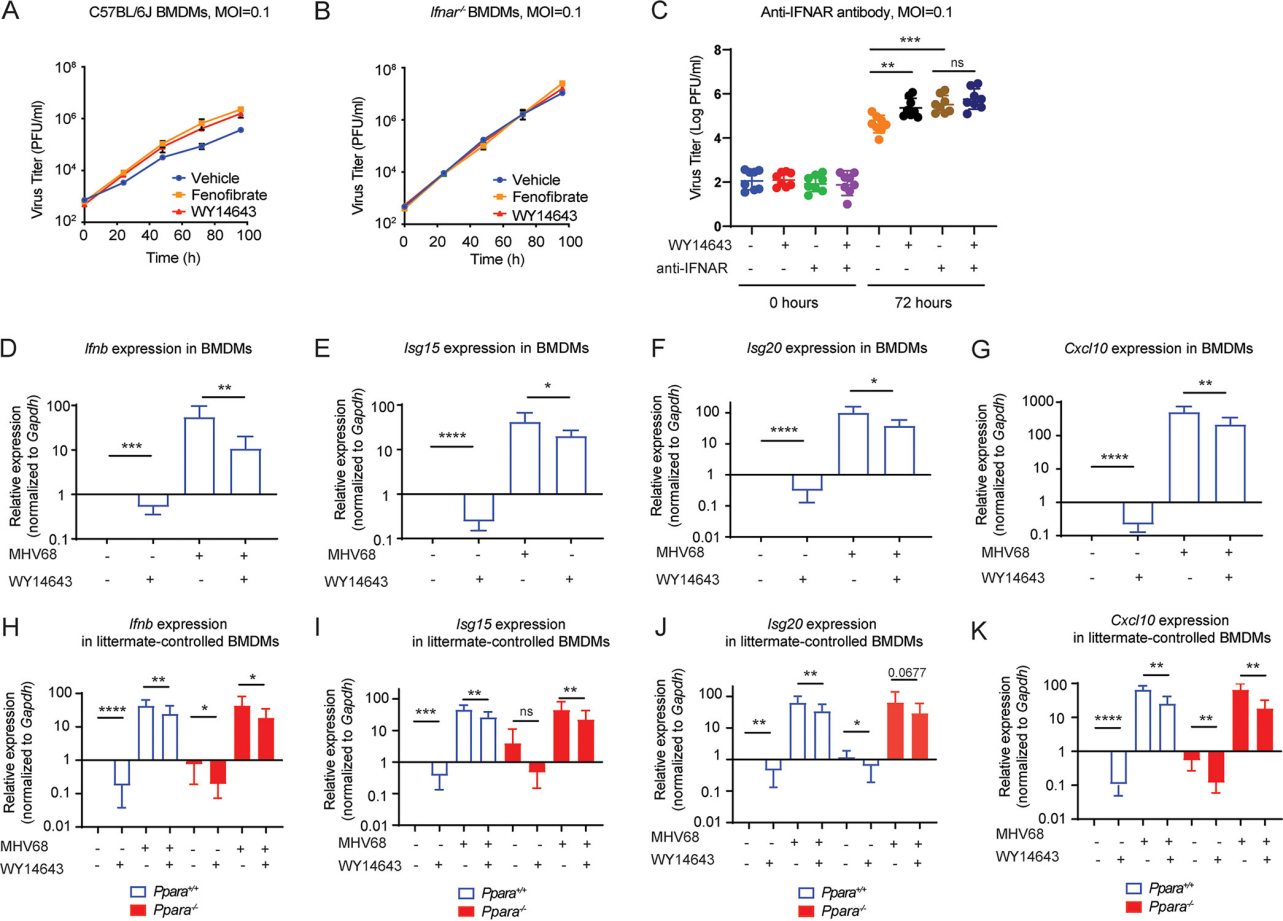
PPARα agonists suppress type I IFN. A–B. Virus replication in C57BL/6J (A) and *Ifnar^−/−^* (B) BMDMs treated with WY14643 and fenofibrate. Cells were pretreated with a vehicle control or an agonist for 16 h prior to infection with MHV68 (MOI = 0.1, *n* = 3). Viral growth was measured over 96 h by plaque assay on 3T12 cells. (C) Virus replication (MOI = 0.1) in C57BL/67 BMDMs treated with WY14643 and anti-IFNAR antibody. Cells were pretreated with DMSO or WY14643 along with 5 μg/mL anti-IFNAR antibody (clone MAR1-5A3, Biolegend number 127312) or an IgG isotype control, then infected. Cells were collected at 0 h and 72 h postinfection. Viral growth was measured by plaque assay on 3T12 cells, and data are shown as the geometric mean ± geometric SD of 8 independent experiments. (D–G) BMDMs from C57BL/6J mice were pretreated with vehicle or WY14643 for 16 h prior to infection with MHV68. Then, RT-qPCR was performed to quantify transcripts of *Ifnb* (*n* = 8), *Isg20* (*n* = 7), *Isg15* (*n* = 6), and *Cxcl10* (*n* = 5) before and 6 h after MHV68 infection. Relative expression of these genes is shown normalized to *Gapdh*. (H–K) BMDMs from littermate-controlled *Ppara^+/+^* or *Ppara^−/−^* mice were pretreated with vehicle or WY14643 for 16 h prior to infection with MHV68. Then, RT-qPCR was performed to quantify transcripts of *Ifnb* (*n* = 5), *Isg20* (*n* = 6), *Isg15* (*n* = 7), and *Cxcl10* (*n* = 6) 6 h after MHV68 infection. Relative expression of these genes is shown normalized to *Gapdh*. (C–K) Data are all shown as the mean ± SD; *, *P* < 0.05; **, *P* < 0.01; ***, *P* < 0.001; ****, *P* < 0.0001. The distribution of RT-qPCR data were checked and found to follow a normal distribution. Plaque assay data were found to follow a lognormal distribution and log-transformed before analysis with one-way ANOVA and Tukey’s multiple-comparison test. RNA transcripts from vehicle and agonist-treated cells were compared with one-tailed paired *t* tests.

We next asked whether the increase in MHV68 replication we observed with WY14643 required the type I interferon receptor, which is important for controlling MHV68 replication (32, 33). We first tested this question with a genetic knockout experiment, isolating BMDMs from *Ifnar*^−/−^ and C57BL/6J mice. The cells were treated as before with WY14643, fenofibrate, or a control and then infected with MHV68 at a low MOI (MOI = 0.1), such that increases in virus replication due to WY14643 treatment could be observed during the multistep growth curve. As expected, WY14643 and fenofibrate increased virus replication in wild-type cells (Fig. 4A). MHV68 also predictably replicated to a higher level in *Ifnar*^−/−^ cells compared to the wild-type cells (Fig. 4B). In contrast to wild-type cells, agonist treatment did not further increase virus replication in *Ifnar^−/−^* BMDMs (Fig. 4B), suggesting that WY14643 and fenofibrate effects depend on type I interferon receptor signaling.

**FIG 5:**
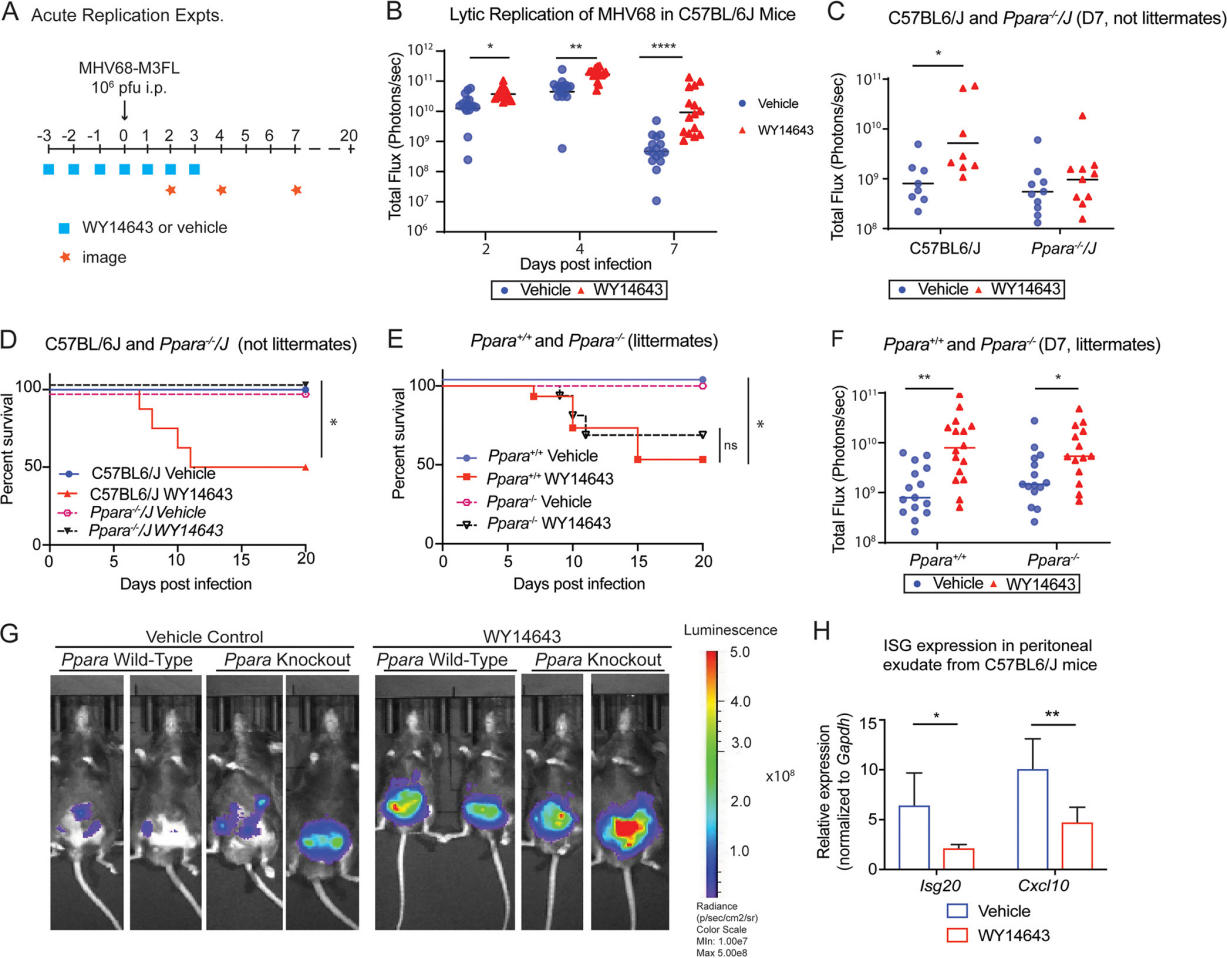
Littermate controls reveal that WY14643 increases viral replication and causes mortality independent of PPARα *in vivo.* (A) The procedure for measuring acute replication of MHV68 in mice. Mice were injected intraperitoneally with either vehicle control (15% HS15 in normal saline) or WY14643 (100 mg drug in HS15 and normal saline per mouse kg) for 7 days, starting 3 days before infection. Mice were infected intraperitoneally with MHV68-M3FL at dose of 10^6^ PFU. Acute replication of virus was measured at day 2, day 4, and day 7 after infection using an IVIS Lumina III imager. Survival of mice was monitored until 20 days after infection. (B) Acute replication of MHV68 over time in C57BL/6J mice measured by luminescent intensity within a region of interest. Mice were treated with vehicle (*n* = 16) or WY14643 (*n* = 15). The data shown are the pool of 3 independent experiments. Dots represent individual animals. (C) Acute replication of MHV68 in C57BL/6J or *Ppara^−/−^/J* mice treated with vehicle control or WY14643 7 days after infection as measured by luminescent intensity. Dots represent individual animals. Data are shown as the pool of 2 independent experiments. (D) Survival rates of mice infected with MHV68. C57BL/6J (*n* = 16) or *Ppara^−/−^/J* (*n* = 20) mice were treated with either a vehicle control or WY14643 and infected with MHV68. Data are shown as the pool of 2 independent experiments. *, *P* < 0.05 by log-rank (Mantel-Cox) test. (E) Survival rates of littermate-controlled mice infected with MHV68. *Ppara^+/+^* (*n* = 30) and *Ppara^−/−^* (*n* = 31) mice were treated with either vehicle control or WY14643, and infected with MHV68. Data are shown as the pool of 3 independent experiments. *, *P* < 0.05 by log-rank (Mantel-Cox) test. (F) Acute replication of MHV68 in littermate-controlled *Ppara^+/+^* (*n* = 30) or *Ppara^−/−^* (*n* = 31) mice measured by luminescent intensity within a region of interest. Dots represent individual animals. The data are shown as the pool of 3 independent experiments. (G) Representative images of littermate-controlled mice taken with the IVIS Lumina III on day 7. The images shown here are on a uniform scale measuring total flux (photons/second). (H) Peritoneal exudate cells were collected from vehicle or WY14643-treated C57BL/6J mice (*n* = 4) 2 days after infection with MHV68 as described in panel A. Relative expression of *Isg20* and *Cxcl10* was quantitated and normalized to *Gapdh*. Data represent 2 independent experiments. Transcripts from peritoneal exudate cells were compared with one-tailed unpaired *t* tests. Data all shown as mean ± SD; *, *P* < 0.05, **, *P* < 0.01, ***, *P* < 0.001, ****, *P* < 0.0001. *In vivo* imaging data were found to follow a lognormal distribution. They were analyzed with 2-way repeated measures ANOVA tests and Tukey’s multiple-comparison tests after log-transformation. The graphed data are untransformed. Survival data were analyzed with Mantel-Cox tests.

To confirm these results, we measured viral replication after blocking IFNAR in C57BL/6J BMDMs with an anti-IFNAR antibody. At 72 hours postinfection, WY14643 treatment increased virus replication compared to the isotype control. Consistent with the results in Fig. 5A and 5B, treatment with the anti-IFNAR antibody increased viral replication compared to the isotype control. However, combining WY14643 with the anti-IFNAR antibody did not cause an additional increase in virus replication (Fig. 4C), indicating that WY14643-mediated increase in MHV68 replication requires IFNAR.

To clarify the role of type 1 interferon in agonist-driven phenotypes, we used RT-qPCR to examine *Ifnb* and ISG expression. We quantified *Ifnb*, *Isg20*, *Isg15*, *and Cxcl10* transcripts in BMDMs from C57BL/6J mice infected with MHV68 for 6 hours. We saw that MHV68 infection induced the transcription of these genes, and that WY14643 treatment significantly attenuated this effect (Fig. 4D to G). We also noted that WY14643 treatment significantly reduced the transcripts of these genes compared to the control even in the absence of viral infection (Fig. 4D to G).

Repeating these experiments in cells from littermate-controlled *Ppara^+/+^* and *Ppara^−/−^* mice, we confirmed that the effect of WY14643 on *Ifnb*, *Isg15*, and *Isg20* is PPARα-independent (Fig. 4H to K). Notably, we again observed that WY14643 reduced the transcripts of *Ifnb*, *Isg15*, *Isg20,* and *Cxcl10* without infection, though the effect was not significant for *Isg20* in *Ppara^−/−^* cells (Fig. 4H to K). This suggests that WY14643 suppression of *Ifnb* gene and ISG expression is independent of PPARα expression.

### WY14643 increases MHV68 replication and lethality independent to PPAR-α expression.

Because we observed that WY14643 increased viral replication and decreased IFNβ production *in vitro*, we tested whether WY14643 increased viral replication in mice. First, we used C57BL/6J and *Ppara^−/−^/J* mice to test the effects of *in vitro* treatment with WY14643. Mice were injected with WY14643 or a vehicle control for 7 days, starting 3 days prior to infection and continuing for 4 days after infection (Fig. 5A). We chose to pretreat mice with WY14643 by intraperitoneal injection because of our *in vitro* data indicating that pretreatment was necessary (Fig. 2). Using luciferase-tagged MHV68 (MHV68-M3FL), we infected mice intraperitoneally and imaged them over multiple days to measure acute virus replication (31, 34). Although it is not physiological, we chose the intraperitoneal route of infection for MHV68 because it is well characterized to lead to equivalent levels of viral replication and latency compared with intranasal (35). Additionally, we chose intraperitoneal infection because we also used intraperitoneal injection to administer WY14643. We found that C57BL/6J mice treated with WY14643 had increased virus replication compared to vehicle-treated mice (Fig. 5B) over 7 days. This elevated virus replication was absent in *Ppara^−/−^/J* mice treated with WY14643 (Fig. 5C). Surprisingly, even though mice were infected with a dose of MHV68 that does not cause lethality in wild-type mice, C57BL/6J mice treated with WY14643 succumbed to infection (Fig. 5D) at a frequency similar to mice deficient in the type I interferon receptor (32, 33, 36). This lethality effect was not present in the PPARα knockout mice (Fig. 5D). As with our *in vitro* data, these *in vivo* data give the appearance that WY14643 increases lethality and virus replication through its canonical function as an agonist of PPARα.

However, after repeating these experiments with mice interbred in our colony, we no longer observed the PPARα-dependency of WY14643. Littermate-controlled knockout mice treated with WY14643 succumbed to virus infection at a similar rate to wild type littermate controls (Fig. 5E) and also displayed significantly increased virus replication at day 7 (Fig. 5F, G).

To determine whether WY14643 treatment *in vivo* suppressed induction of interferon stimulated genes after MHV68 infection, we pretreated mice with WY14643 or control and infected them with MHV68 intraperitoneally as in Fig. 5A. Two days after infection, we sacrificed the mice and collected peritoneal cells by lavage to measure ISG expression. We found that expression of *Isg20* and *Cxcl10* was decreased in WY14643-treated mice (Fig. 5H). These data support the hypothesis that WY14643 suppressed interferon responses *in vivo*, and that this may contribute to increased replication and lethality with MHV68 infection.

## DISCUSSION

We determined that two agonists of PPARα, WY14643 and fenofibrate, increase herpesvirus replication independently of expression of PPARα. In macrophages, WY14643 and fenofibrate increased replication of both a gamma- and beta-herpesviruses. We determined that WY14643 suppressed type I interferon induction after MHV68 infection. *In vivo*, WY14643 increased MHV68 replication and lethality in infected mice, a strong effect given that MHV68 is rarely fatal. The increase in replication and lethality was not dependent on expression of PPARα, but this phenomenon was only revealed when we used littermate control mice for comparison. When we measured ISG expression in peritoneal cells after intraperitoneal infection, we observed decreased ISG expression. Although we cannot conclude the relative contribution of impaired type I interferon to WY14643-induced increase in MHV68 replication and lethality, these data suggest that WY14643 suppressed type I interferon *in vivo*. Given the importance of type I interferon in controlling acute MHV68 infection, we propose that the inhibition of interferon may be one contributing factor to the lethality phenotype. Importantly, the effects of WY14643 on herpesvirus infection and interferon response are independent of PPARα.

Our results imply that WY14643 increased MHV68 replication in part by decreasing interferon production, but further work will be required to describe a more specific mechanism. We observed that WY14643 treatment reduced the transcription of interferon and interferon-stimulated genes even without virus infection. This was true in both wild type and littermate control *Ppara^−/−^* BMDMs. These data suggest that WY14643 regulates basal interferon expression. We do not think this represents global downregulation of transcription by WY14643, both because of previous reports of transcriptomic analysis after agonist treatments and from our own analysis of global gene expression (data not shown) (37).

Although we did not find a PPARα-dependent function of agonists on MHV68 replication, we have not ruled out the possibility that PPARs play a role in virus replication. Such interactions remain worth investigating. One mechanism by which PPARs could regulate virus replication is through the upregulation of negative regulators of inflammation such as IκB and the soluble IL-1 receptor antagonist (38). A second mechanism is by regulating inflammatory gene expression directly. PPARs decrease NF-κB and AP-1 activities through transrepression, which stabilizes corepressor complexes on inflammatory gene promoters, such as nitric oxide synthetase, IL-1β, and IL-12 (39, 40).

Our results indicate that researchers should be mindful of PPAR-independent immune modulation caused by PPAR agonists. Separating PPARα-dependent and independent effects may be complicated by genetic modifiers and microbiome differences in mouse models, as was the case in our experiments. Initially, we observed a PPARα-dependent phenotype when comparing *Ppara^−/−^/J* and C57BL/6J macrophages. The effects of WY14643 and fenofibrate on virus replication were abolished in *Ppara^−/−^/J* cells, suggesting that their mechanism is through their canonical role as PPARα agonists. However, MHV68 replication was unexpectedly high in vehicle-treated *Ppara^−/−^/J* macrophages compared with the control, leading us to question the validity of the comparison. This anomalous virus replication, along with the illusion of PPARα-dependency, disappeared when we generated and used littermate-controlled *Ppara^+/+^* and *Ppara^−/−^* mice. There are several possible reasons for this. For one, the Jackson Laboratory does report that the *Ppara^−/−^/J* mice have 3 single-nucleotide polymorphism markers that are still of the 129S4/SvJae allele-type, which could contribute to differences in immune response and virus replication. Additionally, microbiome differences between our C57BL/6J and *Ppara^−/−^/J* colonies, which were normalized by interbreeding the two, could have played a role. These results are similar to other reports describing the off-target effects of etomoxir, an inhibitor commonly used to block Cpt1a and fatty acid oxidation (41–43). Our results highlight the importance of using littermate controls when trying to establish genetic dependency.

## MATERIALS AND METHODS

### Animals.

C57BL/6J, B6;129S4-*Pparα*^tm1Gonz^/J (44), and B6.129S2-*Ifnar1^tm1Agt^*/Mmjax (45), were purchased from The Jackson Laboratory. All mice were housed under specific pathogen-free, double-barrier facility at the University of Texas Southwestern Medical Center. Mice were fed autoclaved rodent feed and water. Knockout strains were maintained by breeding homozygous knockout males with homozygous knockout females, producing litters with only the knockout genotype.

To generate littermate controls for the *Ppara^−/−^/J* strain, *Ppara^−/−^/J* mice were bred with C57BL/6J mice, producing heterozygous pups. Heterozygous animals were then bred, producing a mixture of heterozygous, wild-type (*Ppara^+/+^*) and knockout (*Ppara^−/−^*) animals. In addition to standard genotyping, we verified the genetics of the crossed animals with RT-qPCR measuring *Ppara* transcripts (46). In *Ppara^+/+^* cells, we measured low-level but consistent (CT = 33.9 ± 0.8, α = 0.05, *n* = 6) transcripts, which were not detectable (CT = undefined) in *Ppara^−/−^* cells.

For isolation of BMDMs, male mice were used. For *in vivo* imaging and survival curves, a mixture of male and female mice was used. Mice were maintained and used under a protocol approved by UT Southwestern Medical Center Institutional Animal Care and Use Committee (IACUC).

### Chemicals and antibodies.

PPARα agonist WY14643 was purchased from Cayman Chemical. For *in vitro* work PPARα agonist fenofibrate, PPARδ agonist GW501516, and PPARγ agonist rosiglitazone were purchased from Sigma-Aldrich. The polyclonal antibody against MHV68 has been previously described (36) and was generously provided by the Virgin lab. The antibody against IFNAR was purchased from Biolegend (clone MAR1-5A3, Biolegend #127312). The isotype control, Mouse IgG1, κ−isotype, was also purchased from Biolegend (clone MOPC-21, Biolegend number 400112).

### Cell culture.

Bone marrow derived macrophages (BMDMs) were differentiated in DMEM (Corning) with 10% FBS supplemented with 1% glutamine (Corning), 1% HEPES (Corning), and 10% CMG14 supernatant for 7 days (47). 3T12 cells were maintained in DMEM with 5% FBS supplemented with 1% glutamine and 1% HEPES. Cell lines are mycoplasma tested.

### Generation of virus stocks.

Murine gamma-herpesvirus 68 (WUSM stain) was purchased from ATCC. Murine gamma-herpesvirus 68-M3FL was generated as previously reported (34).

### Virus infection.

Fully differentiated BMDMs were seeded on 24-well plates (1.5 × 10^5^ cells per well) or 6-well plate (10^6^ cells per well). Cells were pretreated with either vehicle control (0.1% DMSO) or agonists. Fenofibrate was used at a final concentration 50 μM and WY14643 was used at a final concentration of 200 μM (48) Rosiglitazone was used at a final concentration of 1 μM (49) and GW501516 at 100 nM (50) for 16 hours. The next day, macrophages were infected with MHV68 at multiplicity of infection (MOI) = 5 or 0.1. For MCMV experiments, cells were infected at MOI = 1. After an hour, cells were washed with PBS twice to remove unabsorbed viruses. Then, culture medium containing treatments was added to the wells. For growth curves, samples were collected at 0 h, 24 h, 48 h, 72 h and 96 h after infection and were frozen at −80°C. The titer of virus was determined by plaque assay in 3T12 cells. For flow cytometric analysis, cells were collected 24 h after infection.

For IFNAR-blocking experiments, mature BMDMs were pretreated overnight for 16 hours with anti-IFNAR antibody (5 mg/mL) or isotype control and WY14643 (200 μM) or DMSO control. Cells were infected with MHV68 at MOI = 0.1. After 1 hour, the cells were washed twice with PBS to remove unabsorbed virus. Culture media with the same antibody and drug treatments was added to cells. Samples were taken at 0 h and 72 h postinfection and frozen at −80°C. The titer of virus was determined by plaque assay in 3T12 cells.

### Flow cytometry for MHV68 lytic proteins positive cells.

To determine the percentage of cells expressing lytic proteins of MHV68 infection, cells were harvested 24 hours after infection, and fixed with 2% formaldehyde, blocked with 10% mouse serum and 1% Fc block (CD16/32, BioLegend), then stained with polyclonal rabbit antibody to MHV68 (1:1000) (31, 36), followed by secondary goat anti-rabbit Alexa Fluor-647 (Invitrogen).

### Plaque assay.

The concentration of virus was measured by plaque assay in 3T12 cells. The frozen samples containing viruses were thawed in an incubator. The samples were serially diluted, then added to a monolayer of 3T12 cells. After an hour of absorption, the cells were then covered with 1% methylcellulose. Plates were incubated at 37°C for 7 days, and the plaques were stained with 0.1% crystal violet.

### RT-qPCR.

BMDMs in 6-well plates were either infected with MHV68 at MOI = 5 for 6 hours or treated with DMXAA (10 μ/mL) for 4 hours. RNA was extracted using Qiagen RNeasy minikit (Qiagen) and reverse transcribed into cDNA using SuperScript VILO cDNA Synthesis kit (Thermo Fisher Scientific). Relative quantification of target genes was determined using PowerUp SYBR green Master Mix (Thermo Fisher Scientific) in a QuantStudio 7 Flex real-time PCR system. Primers used for amplifying target genes are listed in Table 1.

**TABLE 1:** QPCR primers for target genes

Primers	Sequence
*Ifnb* forward	CAGCTCCAAGAAAGGACGAAC
*Ifnb* reverse	GGCAGTGTAACTCTTCTGCAT
*Cxcl10* forward	TTAACGTCAGGCCAACAGAG
*Cxcl10* reverse	GAGGGAAACCAGGAAAGATAGG
*Isg15* forward	CAGGACGGTCTTACCCTTTCC
*Isg15* reverse	AGGCTCGCTGCAGTTCTGTAC
*Isg20* forward	CCATGGACTGTGAGATGGTG
*Isg20* reverse	CTCGGGTCGGATGTACTTGT
*Gapdh* forward	GGGTGTGAACCACGAGAAATA
*Gapdh* reverse	GTCATGAGCCCTTCCACAAT
*Ppara* forward	CTATAATTTGCTGTGGAGATCGGC
*Ppara* reverse	GGATGGTTGCTCTGCAGGT

### MHV68 acute replication in mice.

Experiments were carried out using 8–12 weeks old mice under the protocol approved by IACUC. Mice were injected intraperitoneally with either vehicle control (15% HS15 in normal saline) or WY14643 (100 mg/kg) for 1 week starting from 3 days before virus infection. Mice were then infected with MHV68-M3FL at the dose of 10^6^ PFU through intraperitoneal route (31, 34). To quantify virus-encoded luciferase expression, mice were weighed and injected with 150 mg/kg of d-Luciferin (GOLDBIO) immediately prior to imaging using an IVIS Lumina III *In Vivo* Imaging System (PerkinElmer). A region of interest (ROI) was drawn around the abdominal region and applied to all mice. Total flux (photons/second) of the ROI was measured (exposure = 1 sec., F/stop = 1.2, FOV=E, binning=medium, emissions filter=open) using Living Image software (PerkinElmer). Survival of the mice was recorded until 20 days after infection.

### Quantification and statistical analysis.

All data are presented as mean ± SD. Statistical comparisons were performed using GraphPad Prism 9.4 software. *In vitro* gene transcripts were compared using paired one-tailed *t* tests. *Ex vivo* gene transcripts were compared using unpaired one-tailed *t* tests. *In vivo* imaging data were analyzed using one-way ANOVA with Tukey’s multiple comparisons post-test. Statistical significance was set at *P* < 0.05. The numbers of independent replicates (n) are reported in the figure legends.
